# Childhood Lead Poisoning Associated with Gold Ore Processing: a Village-Level Investigation—Zamfara State, Nigeria, October–November 2010

**DOI:** 10.1289/ehp.1104793

**Published:** 2012-07-05

**Authors:** Yi-Chun Lo, Carrie A. Dooyema, Antonio Neri, James Durant, Taran Jefferies, Andrew Medina-Marino, Lori de Ravello, Douglas Thoroughman, Lora Davis, Raymond S. Dankoli, Matthias Y. Samson, Luka M. Ibrahim, Ossai Okechukwu, Nasir T. Umar-Tsafe, Alhassan H. Dama, Mary Jean Brown

**Affiliations:** 1 Epidemic Intelligence Service, Centers for Disease Control and Prevention, Atlanta, Georgia, USA; 2 Healthy Homes and Lead Poisoning Prevention Branch, Division of Emergency and Environmental Health Services, National Center for Environmental Health, Centers for Disease Control and Prevention, Atlanta, Georgia, USA; 3 Agency of Toxic Substances and Disease Registry, Atlanta, Georgia, USA; 4 National Center for Chronic Disease Prevention and Health Promotion, Centers for Disease Control and Prevention, Atlanta, Georgia, USA; 5 Office of Public Health Preparedness and Response, Centers for Disease Control and Prevention, Atlanta, Georgia, USA; 6 One Health Office, Division of High Consequence Pathogens and Pathology, National Center for Emerging and Zoonotic Infectious Diseases, Centers for Disease Control and Prevention, Atlanta, Georgia, USA; 7 Nigeria Field Epidemiology and Laboratory Training Program, Abuja, Nigeria; 8 Blood Lead and Inorganic Metals Laboratory, Ministry of Health, Gusau, Zamfara State, Nigeria; 9 Zamfara State Rapid Response Team on Lead Poisoning Outbreak, Ministry of Health, Gusau, Zamfara State, Nigeria

**Keywords:** environmental health, lead poisoning

## Abstract

Background: During May–June 2010, a childhood lead poisoning outbreak related to gold ore processing was confirmed in two villages in Zamfara State, Nigeria. During June–September of that year, villages with suspected or confirmed childhood lead poisoning continued to be identified in Zamfara State.

Objectives: We investigated the extent of childhood lead poisoning [≥ 1 child with a blood lead level (BLL) ≥ 10 µg/dL] and lead contamination (≥ 1 soil/dust sample with a lead level > 400 parts per million) among villages in Zamfara State and identified villages that should be prioritized for urgent interventions.

Methods: We used chain-referral sampling to identify villages of interest, defined as villages suspected of participation in gold ore processing during the previous 12 months. We interviewed villagers, determined BLLs among children < 5 years of age, and analyzed soil/dust from public areas and homes for lead.

Results: We identified 131 villages of interest and visited 74 (56%) villages in three local government areas. Fifty-four (77%) of 70 villages that completed the survey reported gold ore processing. Ore-processing villages were more likely to have ≥ 1 child < 5 years of age with lead poisoning (68% vs. 50%, *p* = 0.17) or death following convulsions (74% vs. 44%, *p* = 0.02). Soil/dust contamination and BLL ≥ 45 µg/dL were identified in ore-processing villages only [50% (*p* < 0.001) and 15% (*p* = 0.22), respectively]. The odds of childhood lead poisoning or lead contamination was 3.5 times as high in ore-processing villages than the other villages (95% confidence interval: 1.1, 11.3).

Conclusion: Childhood lead poisoning and lead contamination were widespread in surveyed areas, particularly among villages that had processed ore recently. Urgent interventions are required to reduce lead exposure, morbidity, and mortality in affected communities.

Lead is highly toxic and can cause damage to the brain, kidneys, bone marrow, and other body systems in humans, especially among young children (Needleman 2004). Lead exposure among children is associated with developmental problems including impaired cognitive function, reduced intelligence, impaired hearing, and reduced stature; no toxicologically safe blood lead level (BLL) has been identified (Canfield et al. 2003; Jusko et al. 2008). At high BLLs, lead can cause convulsions, coma, and death (Needleman 2004). In the United States, the Centers for Disease Control and Prevention (CDC) defines an elevated child BLL as a BLL ≥ 10 µg/dL for initiating public health actions, and recommends that children with BLLs ≥ 45 µg/dL receive intensive medical management and chelation therapy (CDC 2002).

Among developing countries, major sources of childhood lead poisoning include lead mining and smelting, paint, leaded gasoline, battery recycling, and traditional medicines (Falk 2003; Meyer et al. 2008). Exposure to lead ore dust through ingestion or inhalation can result in high BLLs in children. A limited number of studies have documented environmental lead contamination or adults with elevated BLLs in small-scale gold ore–processing communities (Appleton et al. 2001; Betancourt et al. 2005; Odumo et al. 2010; Ogala et al. 2002). However, childhood lead poisoning was not reported to be associated with gold ore processing in these studies.

Zamfara State is located in northwestern Nigeria and has an estimated population of 3.6 million people, and approximately 20% are children < 5 years of age (Joint United Nations Environment Programme/Office for the Coordination of Humanitarian Affairs Environment Unit 2010). Although farming is the major livelihood in Zamfara State, gold ore processing increasingly constitutes an important income source in selected areas. During routine meningitis surveillance in Zamfara State conducted during February–April 2010, Médecins Sans Frontières (MSF) and local public health officials identified > 200 children < 5 years of age with convulsions during the previous 3 months among four villages. Approximately 40 of these children were reported to have died. Environmental causes were suspected because of a recent increase in gold ore–processing activities in the region. Diagnostic tests on eight symptomatic children revealed BLLs of 168–370 µg/dL, levels known to be fatal in children. The unprecedented level of morbidity and mortality raised suspicion about other concomitant diseases such as malaria and bacterial infections; however, laboratory tests failed to demonstrate microorganisms in most patients, and the illnesses did not respond to antimalarials and empiric antibiotics. During May 2010, the Nigerian Federal Ministry of Health assembled a multidisciplinary team consisting of representatives from the Nigerian Field Epidemiology and Laboratory Training Program (NFELTP), Zamfara State Ministry of Health, the CDC, and the World Health Organization (WHO) to join MSF in investigating the outbreak (CDC 2010).

During May–June 2010, the team surveyed the two most-affected villages and confirmed lead poisoning as the cause of the outbreak (CDC 2010; Dooyema et al. 2012). Among these two villages, 25% of children < 5 years of age had died during the previous 12 months, and 82% of these had experienced convulsions before death. All of the 204 children < 5 years of age who were tested had BLLs ≥ 10 µg/dL, and 97% had BLLs ≥ 45 µg/dL. Lead-rich gold ore was identified in both villages. Gold ore–processing activities had begun during the previous 12 months inside a majority of family compounds. Two-thirds of households reported processing gold ore inside family compounds, and soil-lead levels in 85% of family compounds exceeded the U.S. Environmental Protection Agency (EPA) soil-lead standard [400 parts per million (ppm)] for areas of bare soil where children play (U.S. EPA 2003). Factors associated with child mortality in the two surveyed villages were the child’s age, maternal participation in ore processing, and environmental factors such as primary water source type and soil-lead level of the family compound (Dooyema et al. 2012). The investigation concluded that 118 child fatalities were strongly associated with gold ore processing.

During June 2010, the Nigerian Federal Ministry of Health granted expedited approval for therapeutic use of the oral chelating agent meso-2,3-dimercaptosuccinic acid in Nigeria. In response to this public health emergency, the federal government of Nigeria, the Zamfara State government, and international stakeholders including the WHO, the United Nations International Children’s Fund, MSF, TerraGraphics Environmental Engineering, Inc., and CDC jointly implemented control measures, including provision of oral chelation therapy, remediation of lead-contaminated family compounds, public health messaging about lead poisoning, and social mobilization activities to advise villagers to move gold ore processing out of family compounds and villages. Efforts of stakeholders during June–September 2010 resulted in identification of childhood lead poisoning in five additional villages in Zamfara State. By September 2010, > 350 cases of childhood lead poisoning were confirmed. However, local public health professionals in Zamfara State continued to identify additional villages with childhood convulsions and deaths that were suspicious for lead poisoning but not laboratory confirmed. At that time, stakeholders were unable to quantify the number of affected villages. Therefore, the Nigerian Federal Ministry of Health requested that the CDC conduct a follow-up investigation. In contrast to the door-to-door survey among two affected villages conducted during May–June 2010 (Dooyema et al. 2012), this investigation was designed as a rapid assessment to investigate the geographic extent of childhood lead poisoning and lead contamination among villages in Zamfara State and to identify villages that should be prioritized for lead hazard abatement and other urgent interventions.

## Methods

*Setting and sampling of villages***.** Zamfara State has 14 local government areas (LGAs). Each LGA, with an area ranging from 674 to 6,654 km^2^, contains multiple villages within its jurisdiction. A village typically consists of earthen-walled compounds containing open-air dwellings with bare compacted soil floors. Each compound houses at least 1 family. Each village has a chief who interacts with the corresponding local government and traditional leadership system. Census and vital statistics data were unavailable at the village level.

We used chain-referral sampling to identify villages of interest, defined as villages suspected of participation in any gold ore–processing activity during the previous 12 months (October 2009–October 2010) in Zamfara State (Salganik and Heckathorn, 2004). We used chain-referral sampling rather than random sampling to facilitate rapid identification of potentially affected villages. Our chain-referral sampling technique involved an iterative, cyclical process to identify villages of interest. After advocacy visits to state government officials, we visited traditional leaders, local government officials, and stakeholders in the response efforts, asked them to provide a list of villages in their jurisdiction that likely participated in gold ore processing, and developed an aggregate village list. During village visits, we asked villagers to list other villages in their area that likely participated in gold ore processing, added these names to the aggregate list, and repeated the process in subsequent villages. The cycle was repeated during the 5-week operation period (15 October–18 November 2010) to compile a comprehensive list of villages of interest.

*Interviews.* Field investigations were conducted by members from the CDC, NFELTP, and Zamfara State Ministry of Health. Each field team consisted of three to five members and was assigned particular villages of interest. Local traditional leaders designated one to two representatives to accompany and introduce the team to each village. Teams asked the village chief to hold an informal village-wide meeting, informed assembled villagers (predominantly male adults because of cultural norms) of the purpose of the visit, and administered the questionnaire in the local language by interviewing villagers collectively, including the village chief. We used a village-wide meeting instead of only interviewing the village chief because the chief might not know all of the answers or might be reluctant to disclose gold ore–processing activities for fear of legal or economic consequences. The meeting allowed all participating villagers to contribute answers. If villagers disagreed about an answer, the interviewer asked for a consensus decision and recorded the final answer agreed to. Responses were entered into an Epi-Info software version 3.5.1 (CDC, Atlanta, GA, USA) electronic database.

This investigation was conducted in accordance with the Declaration of Helsinki, developed by the World Medical Association (World Medical Association 2008). The Human Subjects Review Board at the CDC determined that this investigation was a public health response that was exempt from full institutional review board review.

*Questionnaire instrument.* We developed a questionnaire to collect information at the village level on the following: estimated number of people and compounds; presence and estimated number of children < 5 years of age who had a history of convulsions during the previous 12 months; presence and estimated number of children < 5 years of age who had a history of convulsions and had died during the previous 12 months; presence and types of gold ore–processing activities during the previous 12 months; and other villages likely to have participated in gold ore processing. Gold ore processing consists of a limited number of core activities [e.g., breaking rock, grinding, washing, drying, amalgamation (using mercury), and melting]. Definitions of these activities have been described previously (Dooyema et al. 2012). Villages might not have participated in all of the ore-processing activities. Villages that reported any gold ore–processing activity were asked to indicate where the ore had been mined.

*Blood collection and analysis.* Because our objective was to rapidly identify and prioritize villages with childhood lead poisoning for interventions, we used the following hierarchy to select approximately five children 2 months to 5 years of age in each village of interest for blood collection: First, we asked to collect blood samples from children who lived in compounds of villagers who were actively participating in gold ore processing. We did not give first priority to sick children in ore-processing compounds because we did not want to oversample children with malaria and other infectious diseases that are endemic in the area. If no villagers actively participated in gold ore processing, the team first requested blood sampling from sick children, preferentially children with a history of convulsions. Finally, if no children met these criteria after repeat inquiries to the villagers, the team then requested blood samples from any five children ages 2 months to 5 years. Teams obtained informed consent from a child’s parents before drawing blood.

Product lots of all blood collection supplies used in this investigation were prescreened for lead contamination by CDC laboratories. Blood collection supplies were stored in plastic gallon-size bags before blood collection to prevent lead contamination in the field. Venipuncture sites were thoroughly cleaned with alcohol wipes, and trained team members collected 1–3 mL of venous blood from each child in a tube containing ethylenediamine tetraacetic acid. Blood samples were analyzed within 8–72 hr of collection at the Blood Lead and Inorganic Metals Laboratory in Gusau, Zamfara, using a portable blood lead analyzer, LeadCare II® (Magellan Biosciences, Chelmsford, MA, USA), which can quantify BLLs registering 3.3–65 µg/dL with an average bias from reference of 4.7–5.0% (Freeney and Zink 2007).

*Environmental specimen collection and analysis.* Teams collected two types of environmental samples for lead testing. In each compound where a child’s blood was sampled, household dust samples were collected from piles created by sweeping or scooping loose floor dust, preferentially from areas where children ate or slept. In each village, surface soil was collected from at least one public area. To increase the chance of identifying lead contamination, the team preferentially sampled soil from public areas near a large centrally located shade tree, a typical location for breaking rocks (an early ore-processing activity), or near accessible water sites that might have been used for washing ore. Among villages that reported having processed ore, the team identified ore-processing sites by conducting a walk-through visual assessment and interviewing villagers who were familiar with the ore-processing practices of the village. The team collected surface soil samples from as many as five ore-processing locations, with priority given to grinding, washing, and drying sites because these activities are most likely to generate dust or waste and contaminate the soil (Dooyema et al. 2012).

All soil and household dust samples were placed in separate, clean, zipped plastic bags and later analyzed at the Blood Lead and Inorganic Metals Laboratory in Gusau, Zamfara, for lead levels using a portable, hand-held X-ray fluorescence spectrometer (NITON^®^ XL3; Thermo Fisher Scientific, Billerica, MA, USA). The minimum detection limit for lead in the soil and dust samples ranged from 15 to 20 ppm. Consistent with U.S. EPA guidelines on initial assessment of lead-contaminated residential properties, the team categorized soil and dust lead levels using 400 ppm and 1,200 ppm as thresholds to prioritize response actions (U.S. EPA 2003).

*Outcome and exposure variables.* Village-level outcome variables were examined as follows: ≥ 1 child < 5 years of age who had convulsions during the previous 12 months; ≥ 1 child < 5 years of age who had convulsions and died during the previous 12 months; ≥ 1 child < 5 years of age with a BLL ≥ 10 µg/dL; ≥ 1 child < 5 years of age with a BLL ≥ 45 µg/dL; ≥ 1 soil/dust sample with a lead level > 400 ppm; and ≥ 1 soil/dust sample with a lead level > 1,200 ppm. A village with childhood lead poisoning was defined as a village with ≥ 1 child with a BLL ≥ 10 µg/dL. A village with lead contamination was defined as a village with ≥ 1 soil/dust sample with a lead level > 400 ppm. Villages that reported participation in any gold ore–processing activity during the previous 12 months were classified as ore-processing villages. Villages that reported no participation in gold ore–processing activity during the previous 12 months were classified as non-ore-processing villages.

*Statistical analysis.* Variables were analyzed using SAS version 9.2^®^ (SAS Institute Inc., Cary, NC, USA). Medians and ranges were calculated for population estimates with skewed distributions. The village was the unit of analysis, rather than the children or soil samples. We conducted bivariate analyses using chi-square analysis or Fisher’s exact test to compare categorical outcome variables at the village level between ore-processing villages and non-ore-processing villages, and estimated unadjusted odds ratios (OR) and 95% confidence intervals (CI). All comparisons were two-tailed, and a *p*-value < 0.05 was considered statistically significant.

## Results

**Figure 1:**
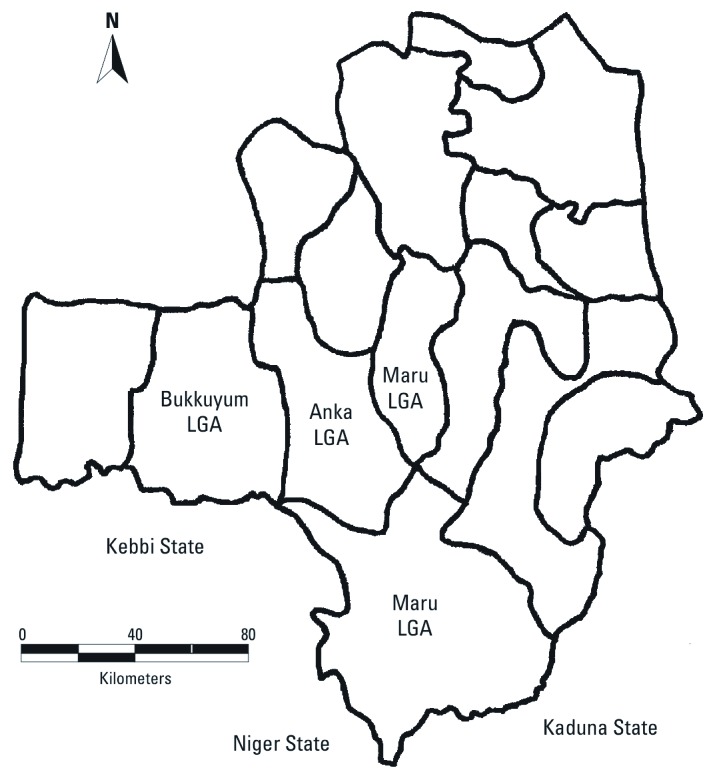
Map of Zamfara state by administrative boundaries of Local Government Areas (LGA), highlighting Anka, Bukkuyum, and Maru LGAs and their neighboring states. The jurisdiction of Maru LGA comprises discrete northern and southern areas.

We identified 131 villages of interest located in 10 of the 14 LGAs in Zamfara State. Because of time and logistic constraints, the team focused on the 114 (87%) villages of interest located within three LGAs: Anka, Bukkuyum, and Maru. All three LGAs are located in the southwestern region of Zamfara State and are bordered by Kebbi, Niger, and Kaduna States (Figure 1).

During 15 October–18 November 2010, we visited 74 of 114 (65%) villages of interest, including 32 of 50 (64%) villages of interest in Anka LGA, 4 of 9 (44%) villages of interest in Bukkuyum LGA, and 38 of 55 (69%) villages in Maru LGA. The 40 villages not visited were either inaccessible because of poor road conditions (13 villages) or were too remote to reach in 1 day (27 villages). Among the 74 villages visited, 70 (95%) completed the survey and were included for analysis.

Among these 70 villages, the estimated median population was 1,800/village (range, 150–13,000), and the estimated median number of family compounds was 100/village (range: 11–1,000). The estimated median number of children < 5 years of age with convulsions during the previous 12 months was 19/village (range, 0–200), and the median number of children who had died following convulsions was 5/village (range, 0–100). Venous blood was collected from 314 children in the 70 villages (median/village 5; range, 1–8). The median age of the children sampled was 3 years (range, 6 months–4 years), and BLLs among the sampled children ranged from < 3.3 to > 65 µg/dL). Lead levels in soil/dust samples collected from the 70 villages ranged from undetectable to 87,293 ppm.

Fifty-four (77%) of the 70 villages reported participation in any gold ore–processing activity during the previous 12 months and thus were classified as ore-processing villages. The most common gold ore–processing activities were breaking rocks (69%), washing (70%), and drying (69%) (Table 1). Ore-processing villages identified 84 sources of ore that were geographically dispersed among the three surveyed LGAs in Zamfara state, and in Kebbi, Niger, and Kaduna states (data not shown).

**Table 1:** Gold ore–processing activities in villages of interest during the previous 12 months [n (%)].

Activity	Participating villages (*n* = 70)
Breaking rocks	48 (69)
Grinding	29 (41)
Washing	49 (70)
Drying	48 (69)
Amalgamation and melting	43 (61)
≥ 1 of the above activities	54 (77)

**Figure 2:**
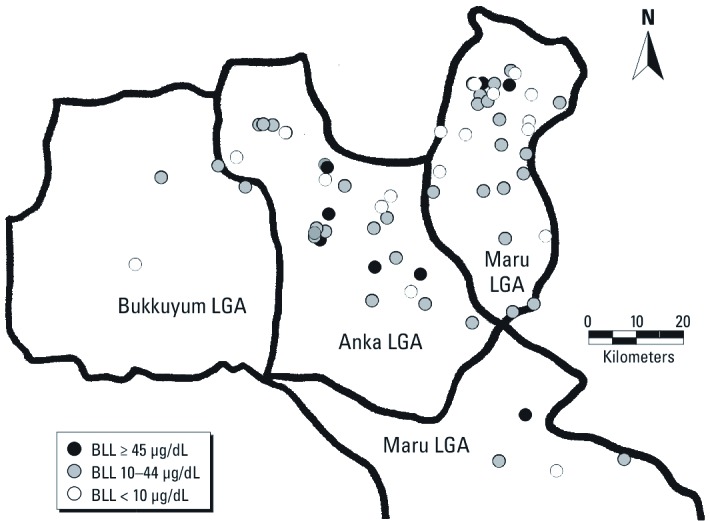
Distribution of surveyed villages by BLLs.

**Figure 3:**
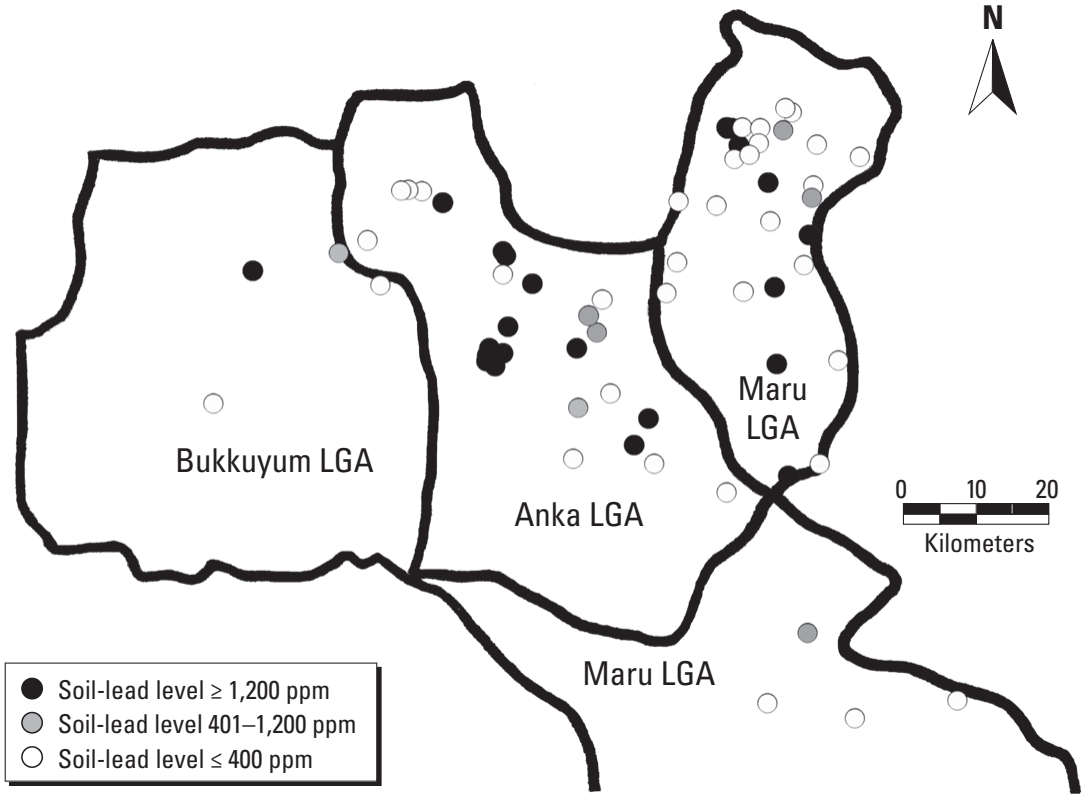
Distribution of surveyed villages by soil lead levels.

Villages with childhood lead poisoning and lead contamination were widely distributed in the three LGAs (Figures 2 and 3). Ore-processing villages were more likely than the other villages to report that ≥ 1 child < 5 years of age had convulsions during the previous 12 months (85% vs. 50%, *p* = 0.003) and that ≥ 1 child had died following convulsions (74% vs. 44%, *p* = 0.02) (Table 2). Ore-processing villages also were more likely to have ≥ 1 child with a BLL ≥ 10 µg/dL (68% vs. 50%, *p* = 0.17), and children with a BLL ≥ 45 µg/dL were identified in ore-processing villages only (8 of 54 villages vs. 0 of 16, *p* = 0.22). Half of the ore-processing villages had ≥ 1 soil/dust sample with a lead level > 400 ppm compared with none of the other villages (*p* < 0.001). Twenty ore-processing villages (37%) had ≥ 1 soil/dust sample with a lead level > 1,200 ppm. Fifty (71%) villages had childhood lead poisoning, lead contamination, or both, including 42 of 54 ore-processing villages and 8 of 16 non-ore-processing villages (OR = 3.5; 95% CI: 1.1, 11.3; *p* = 0.03). Among the 27 ore-processing villages with soil/dust lead contamination, 22 (81%) had ≥ 1 child with a BLL ≥ 10 µg/dL and 8 (30%) had ≥ 1 child with a BLL ≥ 45 µg/dL. In comparison, among the 43 villages without soil/dust samples with lead levels > 400 ppm (27 ore-processing villages and 16 of 16 non-ore-processing villages), 23 (53%) had ≥ 1 child with a BLL ≥ 10 µg/dL and none had ≥ 1 child with a BLL ≥ 45 µg/dL.

**Table 2:** Comparisons of characteristics between ore-processing villages and non-ore-processing villages.

Characteristic	Ore-processing villages			OR (95% CI)	*p*-Value
	Yes (*n* = 54) *n* (%)	No (*n* = 16) *n* (%)			
≥ 1 child < 5 years of age who had convulsions during the previous 12 months	46 (85)	8 (50)	5.8 (1.7, 19.8)	0.003
≥ 1 child < 5 years of age who had convulsions and died during the previous 12 months	40 (74)	7 (44)	3.7 (1.2, 11.7)	0.02
≥ 1 child < 5 years of age with a BLL ≥ 10 µg/dL	37 (68)	8 (50)	2.2 (0.7, 6.8)	0.17
≥ 1 child < 5 years of age with a BLL ≥ 45 µg/dL	8 (15)	0 (0)	undefined	0.22
≥ 1 soil/dust sample with a lead level > 400 ppm	27 (50)	0 (0)	undefined	< 0.001
≥ 1 soil/dust sample with a lead level > 1,200 ppm	20 (37)	0 (0)	undefined	0.003
≥ 1 child < 5 years of age with a BLL ≥ 10 µg/dL or ≥ 1 soil/dust sample with a lead level > 400 ppm	42 (78)	8 (50)	3.5 (1.1, 11.3)	0.03

## Discussion

*Findings.* Results of this investigation demonstrated widespread childhood lead poisoning and lead contamination among villages in Anka, Bukkuyum, and Maru LGAs in Zamfara State. The majority of villages surveyed had at least one child < 5 years of age with a BLL ≥ 10 µg/dL, regardless of ore-processing status, and children < 5 years with convulsions and death following convulsions also were reported in both ore-processing and non-ore-processing villages. However, childhood convulsions and deaths and high soil/dust-lead levels were significantly more likely to be found in villages reporting recent gold ore processing than in other villages. Our data support the hypothesis that processing lead-rich gold ore resulted in lead-contaminated villages and homes, lead poisoning among young children, and possibly convulsions and death from severe lead poisoning. Urgent interventions are required to reduce lead exposure, morbidity, and mortality in affected communities.

*Gold mining and lead exposure.* Gold production has increased worldwide concomitant with global demand. The price of gold has increased approximately 360% from 2001 to 2010 (Swenson et al. 2011). Among developing countries, small-scale gold ore processing and production have increasingly been adopted by rural communities. The environmental and health impacts of small-scale gold production are often overlooked. Gold mining and processing are known to cause air and water pollution from arsenic, mercury, and cyanide. Gold processing can also cause mercury poisoning in workers because of direct exposure to liquid or vaporized mercury during ore processing (Swenson et al. 2011). Although lead pollution is not commonly associated with gold mining, studies of small-scale gold mining sites in the Migori gold belt (Kenya) have demonstrated lead, mercury, and arsenic pollution of multiple gold processing sites; recorded soil lead levels ranged from 16 to 14,999 ppm (Odumo et al. 2010; Ogala et al. 2002). A study in Ecuador demonstrated lead, manganese, and mercury pollution of river water near the surveyed small-scale gold-mining sites; approximately 40% of adults from the affected communities had BLLs > 20 µg/dL (Betancourt et al. 2005). The widespread childhood lead poisoning and lead contamination identified during our investigation illustrates the urgent need to monitor and prevent lead pollution from gold ore–processing activities, particularly among small-scale gold mining communities globally.

In Zamfara State, gold mining began in the early 1900s (Nigeria Ministry of Mines and Steel Development 2010a), but our prior investigation in two affected villages showed that most families did not begin gold ore processing until 2009–2010 (Dooyema et al. 2012). Lead was not previously known to be present in this part of the country until high lead levels in gold ore from locations in Zamfara State were identified through recent exploration activities intended to identify new mining sites (Nigeria Ministry of Mines and Steel Development 2010a, 2010b). A substantial number of geographically dispersed locations in Zamfara State and its neighboring states were reported as sources of ore; lead content in ore from these locations remains unknown. Geologic studies are needed to systematically investigate the lead content of gold ore in this region and identify sources of ore that may increase the risk of lead poisoning among miners and their families.

Processing lead-rich ore inside villages leads to lead contamination. Contaminated soil and household dust are likely to be the main sources of lead exposure for children living in ore-processing villages. During this investigation we applied U.S. EPA standards established to protect children from typical soil-lead exposures in the United States to categorize lead levels in the soil and household dust samples (U.S. EPA 2003). However, because of bare soil flooring, unpaved roads, and lack of vegetative cover, children in the surveyed villages are likely to interact with the soil in their environment more closely and frequently than do children in the United States, which may result in increased exposures at soil-lead levels below U.S. EPA thresholds (U.S. EPA 2003). Therefore, further investigation to determine appropriate standards for health-protective soil-lead levels in such communities is warranted. Furthermore, although BLLs ≥ 45 µg/dL and soil-lead levels > 400 ppm were reported exclusively in ore-processing villages, children with BLLs ≥ 10 µg/dL were present in half of the non-ore-processing villages. Leaded gasoline was phased out in Nigeria during 2003 (United Nations Environmental Program 2010), but other potential sources of lead exposure, such as water supplies or traditional medicine, should be investigated in future studies.

*Recommendations.* Villages with high soil-lead levels identified during this investigation should be prioritized for public health messaging and environmental interventions to reduce childhood lead exposure. Because gold ore processing has become an important source of income, banning villagers from conducting any gold ore–processing activity would be impractical and may damage their economic viability. Instead, gold ore–processing activities should be prohibited inside villages and moved to secured locations that are inaccessible to children. Miners and persons who process ore should be educated regarding occupational precautions and protective measures to reduce the risk of harmful lead exposure for themselves and their families. Miners should change their clothes and shoes when entering and leaving worksites to reduce the likelihood of carrying lead-contaminated dust home. Gold ore–processing activities within villages should be monitored and prohibited to prevent further contamination. Radio programs, dramas, and community gatherings are being used to educate villagers about the health effects of lead, sources of lead, and ways to reduce lead exposure in their homes, villages, and worksites. Both ore-processing villages and non-ore-processing villages should receive these messages given the rapid proliferation of gold ore processing in this region.

Villages with high soil lead levels should also be prioritized for environmental interventions. Assessments should include measurement of lead exposure in all public areas and all households in the village, and environmental remediation efforts should include removal of lead-contaminated ore-processing materials, surface soil, and household dust, and replacing removed surface soil with clean soil. During June 2010–March 2011, such environmental remediation was successfully conducted in seven villages (Blacksmith Institute 2011).

Case identification and treatment for lead poisoning should first be extended to villages in which a child with a BLL ≥ 45 µg/dL was identified, followed by villages with childhood lead poisoning, lead contamination, or both. Children with frank lead poisoning will require long-term follow-up and support. Health care providers in this region need to be trained to diagnose and treat lead poisoning, and medical treatment should be coordinated with environmental interventions because chelation therapy alone will be ineffective if children remain in contaminated homes.

Even with remediation efforts and medical treatment, recontamination and recurrent lead poisoning can occur if the source of lead exposure (e.g., ore processing) continues to be present or is reintroduced in villages. State and local authorities should develop and sustain the capacity to institute blood and environmental lead surveillance to identify new cases and affected villages, and monitor BLLs and environmental lead contamination during and after implementation of control measures.

*Limitations.* Villages of interest were identified through a chain-referral sampling process that might not have captured all villages involved with gold ore processing in Zamfara State, and hard-to-reach or inaccessible villages of interest were not investigated. Therefore, we did not fully determine the geographic extent of childhood lead poisoning in Zamfara State. Second, we could only sample approximately five children in each village. Although we oversampled children from homes with gold ore processing and sick children to increase the likelihood of detecting elevated BLLs, the number of villages with childhood lead poisoning might have been underestimated. Third, we used convulsions and deaths as clinical indicators for lead poisoning because symptoms of lead poisoning are generally nonspecific, and blood lead testing was unavailable at the beginning of the outbreak when the majority of affected children died. However, convulsions and deaths are not sensitive as markers of lead poisoning because they typically occur at BLLs that are higher than the levels detected in most blood samples collected from these villages, and convulsions and death are not specific for lead poisoning because they can result from other locally endemic diseases, including malaria and bacterial meningitis. Finally, we did not assess lead poisoning among children ≥ 5 years of age, adults, or livestock. Older children and adults are also at risk for harmful lead exposure and its adverse health effects, and children may be exposed *in utero* or through breast-feeding if their mothers have elevated BLLs. In addition, villagers may be exposed through consumption of lead-contaminated food or dairy products. Therefore, characterizing the depth and extent of lead poisoning in older children, adults, and livestock is an important topic for further investigation.

## Conclusion

Childhood lead poisoning and lead contamination were widespread in the three surveyed LGAs in Zamfara State. Villages with recent gold ore–processing activity are at increased risk for childhood lead poisoning and lead contamination. We recommend cooperative action among international stakeholders and Nigerian federal, state, and local authorities to eliminate or reduce lead poisoning in this region. Joint efforts are needed to relocate gold ore–processing activities to locations inaccessible to children, fully assess and remediate contaminated villages, communicate health messages and enhance risk awareness in affected communities, sustain screening and medical treatment programs, and continue to identify lead-contaminated villages. Adequate input of resources and sustained commitment from the Nigerian federal, state, and local authorities and international stakeholders are essential to ensure timely and optimal implementation of control measures in response to this unprecedented environmental catastrophe.
